# Phyto-Mediated Photo Catalysed Green Synthesis of Silver Nanoparticles Using *Durio Zibethinus* Seed Extract: Antimicrobial and Cytotoxic Activity and Photocatalytic Applications

**DOI:** 10.3390/molecules23123311

**Published:** 2018-12-13

**Authors:** Samuggam Sumitha, Sethu Vasanthi, Sivadasan Shalini, Suresh V. Chinni, Subash C.B. Gopinath, Periasamy Anbu, Mohammed Baidi Bahari, Rajak Harish, Sathasivam Kathiresan, Veerasamy Ravichandran

**Affiliations:** 1 Faculty of Applied Science, AIMST University, Semeling 8100, Bedong, Kedah, Malaysia; sumithasamuggam@gmail.com (S.S.); v_suresh@aimst.edu.my (S.V.C); kkdjps@gmail.com (S.K); 2 Faculty of Engineering, The University of Nottingham, Semenyih 43500, Selangor, Malaysia; Vasanthi.Sethu@nottingham.edu.my; 3 Department of Pharmacy Practice, KMCH College of Pharmacy, Coimbatore 641035, India; shaliniravichandran11@gmail.com; 4 Faculty of Pharmacy, AIMST University, Semeling 8100, Bedong, Kedah, Malaysia; baidi_bahari@aimst.edu.my; 5 School of Bioprocess Engineering, Universiti Malaysia Perlis, Arau 02600, Perlis, Malaysia; subash@unimap.edu.my; 6 Department of Biological Engineering, Inha University, Incheon 402-751, Korea; anbu25@yahoo.com; 7 SLT Institute of Pharmaceutical Sciences, Guru Ghasidas University, Bilaspur 495009, India; harishdops@yahoo.co.in

**Keywords:** *Durio zibethinus* seed, green synthesis, silver, nanoparticles, antibacterial, cytotoxicity

## Abstract

In the present study, we have developed a green approach for the synthesis of silver nanoparticles (DSAgNPs) using aqueous extract of *Durio zibethinus* seed and determined its antibacterial, photocatalytic and cytotoxic effects. Surface plasmon resonance confirmed the formation of DSAgNPs with a maximum absorbance (λ_max_) of 420 nm. SEM and TEM images revealed DSAgNPs were spherical and rod shaped, with a size range of 20 nm and 75 nm. The zeta potential was found to be −15.41 mV. XRD and EDX analyses confirmed the nature and presence of Ag and AgCl. DSAgNPs showed considerable antibacterial activity, exhibited better cytotoxicity against brine shrimp, and shown better photocatalytic activity against methylene blue. Based on the present research work, it can be concluded that DSAgNPs could be used in the field of water treatment, pharmaceuticals, biomedicine, biosensor and nanotechnology in near future.

## 1. Introduction

Nanoparticles have been extensively explored in the fields of optics, catalysis, engineering, biology, medicine and pharmaceutical sciences because of their unique chemical and physical properties. Different approaches have been practiced for preparing nanoparticles, including chemical or electrochemical methods. Generally, the utilized chemicals or the by-products formed during the process are hazardous that not only increase difficulty in purification stages but these also require high energy for the preparation [1,2]. Apart of the toxicity problems, researchers frequently encounter other major challenges when preparing nanoparticles, such as control of size/shape variation and monodispersity attainment. To address such issues, the use of plant extracts has evolved as one of the most promising options for the synthesis of nanoparticles. The plant constituents act as reducing as well as protective agents for the reduction of metal ions to their corresponding nanoparticles and protect them from aggregation by capping [3]. The key advantages of this method are the absence of complex purification steps and maintenance of microbial cell cultures [4]. As increased interest has been observed in nanotechnology and biotechnology during the last two decades, an increased need for the development of innovative technologies for biosynthesis of nanoparticles has also been noticed [5].

Most of the reported silver nanoparticles synthesis methods are found to be associated with various problems, including lack of stability, growth of crystals and particle aggregation. Silver-based products have been utilized over the centuries to prevent and treat numerous diseases, especially infections, due to strong growth inhibitory and bactericidal effects of silver [6]. Moreover, biosynthesized silver nanoparticles have also indicated antimicrobial activities [7,8,9], anticancer activity [10], anti-inflammatory effects [11], anti-viral activities [12], anti-angiogenesis activities [13] and antidiabetic activities [14].

*Durio zibethinus* (durian) plant, a member of Malvaceae family, has been reported to possess antioxidant, anti-diabetic, anti-hyperlipidemic and anti-proliferative activities [15,16]. Moreover, the nutritional values and health benefits associated with durian consumption have also been observed [17,18]. Its medicinal properties have collectively been attributed to several bioactive compounds present in durian fruit, such as flavonoids, tannins, polyphenols, carbohydrates, protein, vitamin B, vitamin C, calcium, potassium, iron, manganese, sodium, magnesium, and copper [19,20]. Toledo et al. reported the presence of amino acids [16], and Hokputsa et al. reported saccharides and sterols as essential elements of durian seed [21].

The main objective of our study was to utilize aqueous extract of *Durio zibethinus* fruit waste (seed) for the biosynthesis of silver nanoparticles. Moreover, the antibacterial potential of DSAgNPs against various bacterial strains was evaluated and photocatalytic activities of DSAgNPs were tested against methylene blue. Additionally, brine shrimp cytotoxicity assay was conducted to analyze preliminary toxicity which could be extrapolated for cell-line toxicity and anti-tumor activities [22,23]. The significance of the present study was, for the first time we had utilized the fruit waste (seed) extract of *Durio zibethinus* for the biological reduction of silver nanoparticles using under sunlight, and the synthesized silver nanoparticles showed better antimicrobial, cytotoxic and photocatalytic activity with good stability.

## 2. Results and Discussion

### 2.1. Synthesis of Silver Nanoparticles: Process Optimization

In the present study, green synthesis of silver nanoparticles was achieved by bio-reduction of silver nitrate utilizing *Durio zibethinus* seed aqueous extract as reducing and capping agent. The selection criteria for durian seed was based on several factors, viz. the presence of saccharides, which are responsible for the reduction of Ag^+^ in reaction solution, amino acids for stabilization of the formed silver nanoparticles, and preference in this area of research, and as it is a waste that causes environmental pollution [24,25,26,27,28].

**Figure 1:**
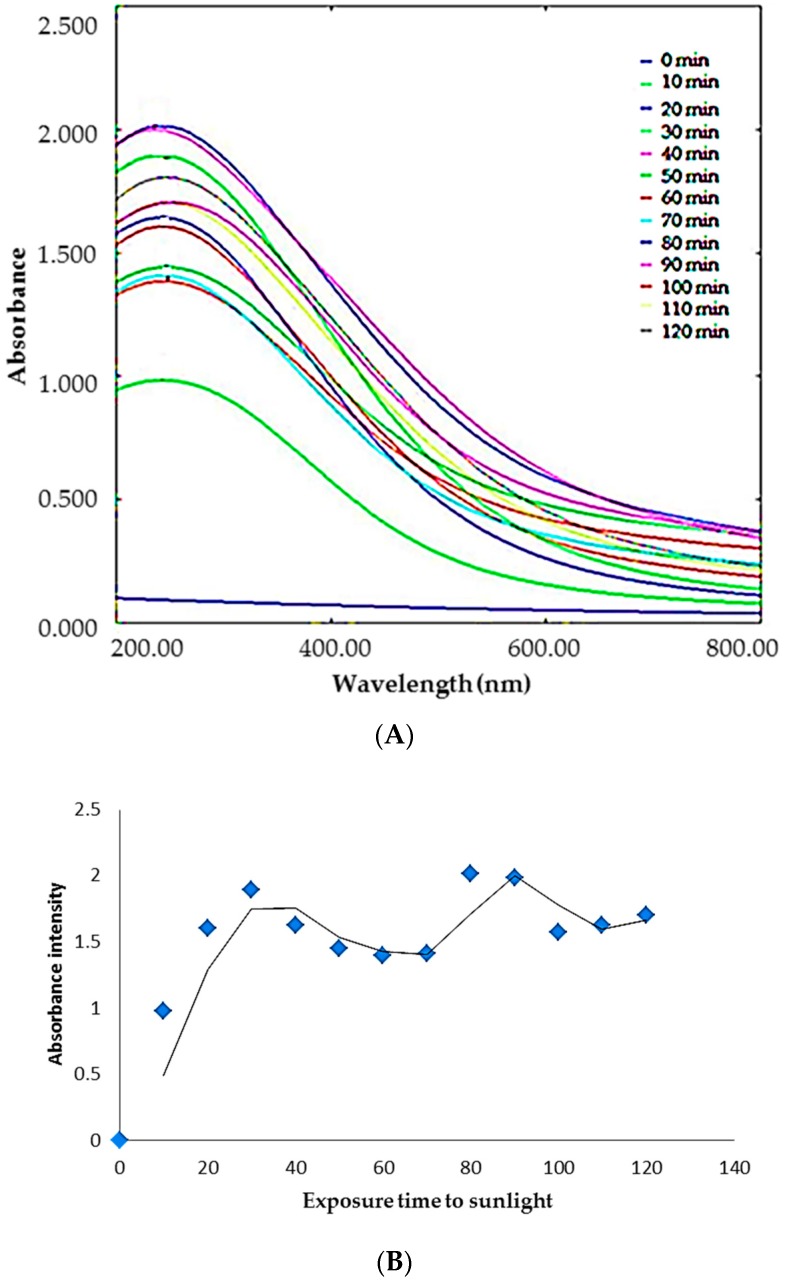
(**A**) UV-visible absorption spectra of synthesized DSAgNPs at different time interval under sunlight, (**B**) Effect of exposure time (min) to sunlight on formation of DSAgNPs.

Aqueous extract of durian seeds was utilized to reduce AgNO_3_ to Ag^0^ where the observed color change from colorless to yellowish brown confirms the reduction. The maximum absorbance exhibited by the reaction solution at 420 nm (Figure 1A) is the characteristic surface plasmon resonance (SPR) peak of DSAgNPs. Factors including size and shape of particles formed could have influenced SPR peak formation [29].

Initially, the reaction was carried out with a fixed concentration of silver nitrate (1 mM) and seed extract (1 mL) at different conditions like room temperature, at 60 °C, in the dark and under sunlight. The reactant solution which was kept at room temperature for 72 h, heated at 60 °C for 1 h and kept in dark for 72 h, did not demonstrate significant color change or absorbance peak in UV-Vis spectral studies.

A significant change in the color and absorbance of the reactant solution was only observed when it was kept in the sunlight (approximately 4000 ± 200 lux) for 10 min. Subsequently, parameters viz. concentration of silver nitrate, time of reaction and volume of durian seed extract required for synthesis of DSAgNPs were optimized. Based on the results, it could be deduced that 1.5 mL extract of 10% durian seed in deionized water, 1.5 mM AgNO_3_ concentration, 1.5:8.5 ratio of seed extract and silver nitrate, and 30 min reaction time in sunlight were found to be the optimized conditions for synthesis of DSAgNPs. The sunlight exposure time for the formation of silver nanoparticles was optimized as 30 min (Figure 1A) since there was a bathochromic and hypochromic shift in the visible absorption spectrum after 30 min which indicates the agglomeration of silver clusters that leads to the formation of larger size particles [30]. Also there was a second minor absorbance peak at a wavelength near about 700 nm at an exposure time of 80 and 90 min, so we have selected 30 min as the optimum time even though the colloidal solution of 80 and 90 min exposure time showed higher absorbance. Figure 1B indicates the uncertainty in silver nanoparticles formation when the solution was exposed to sunlight for longer time.

### 2.2. Mechanism Involving in DSAgNPs Formation

The present study dealt with a waste-mediated silver nanoparticles synthesis using durian seed aqueous extract, which might be instigated by the presence of saccharides and sterols in the extract. In this study, the −OH and O present in side chains of the saccharides might be involved in the reduction, as mentioned by Nadezhda et al. who stated that the −COO−, −OH and O present in hydrolyzed products (galacturonic acid, galactose and arabinose) of pectin fragments were involved in the development of silver nanoparticles [31]. The capping could also be facilitated by amino acids and enzymes present in the seed extract. The possible reason behind this behavior might be the presence of a greater number of photons of a certain wavelength in direct sunlight which catalyse the reducing action by the generation of reducing moieties (e−), thus promoting the formation of AgNPs [30].

### 2.3. Characterization of DSAgNPs

**Figure 2:**
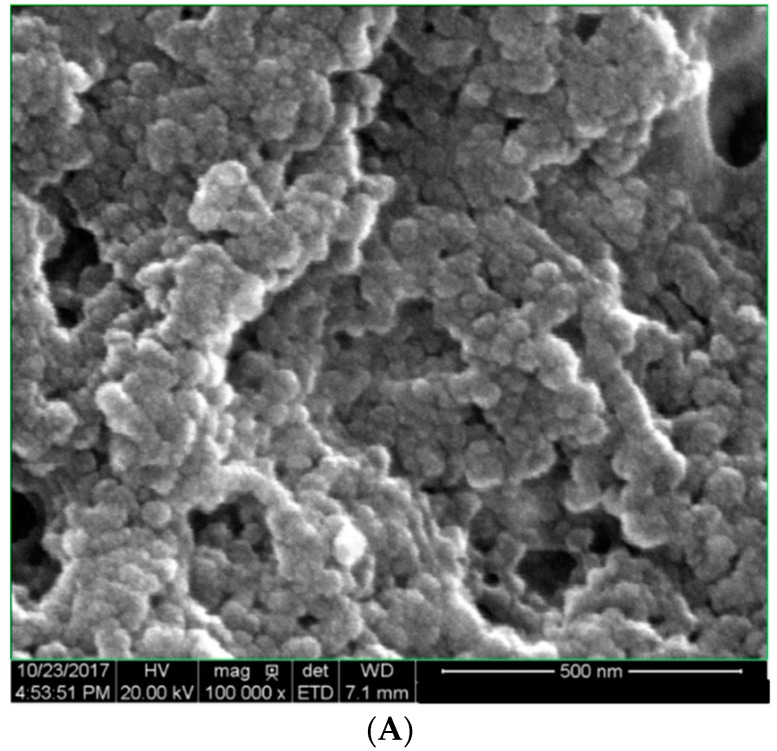

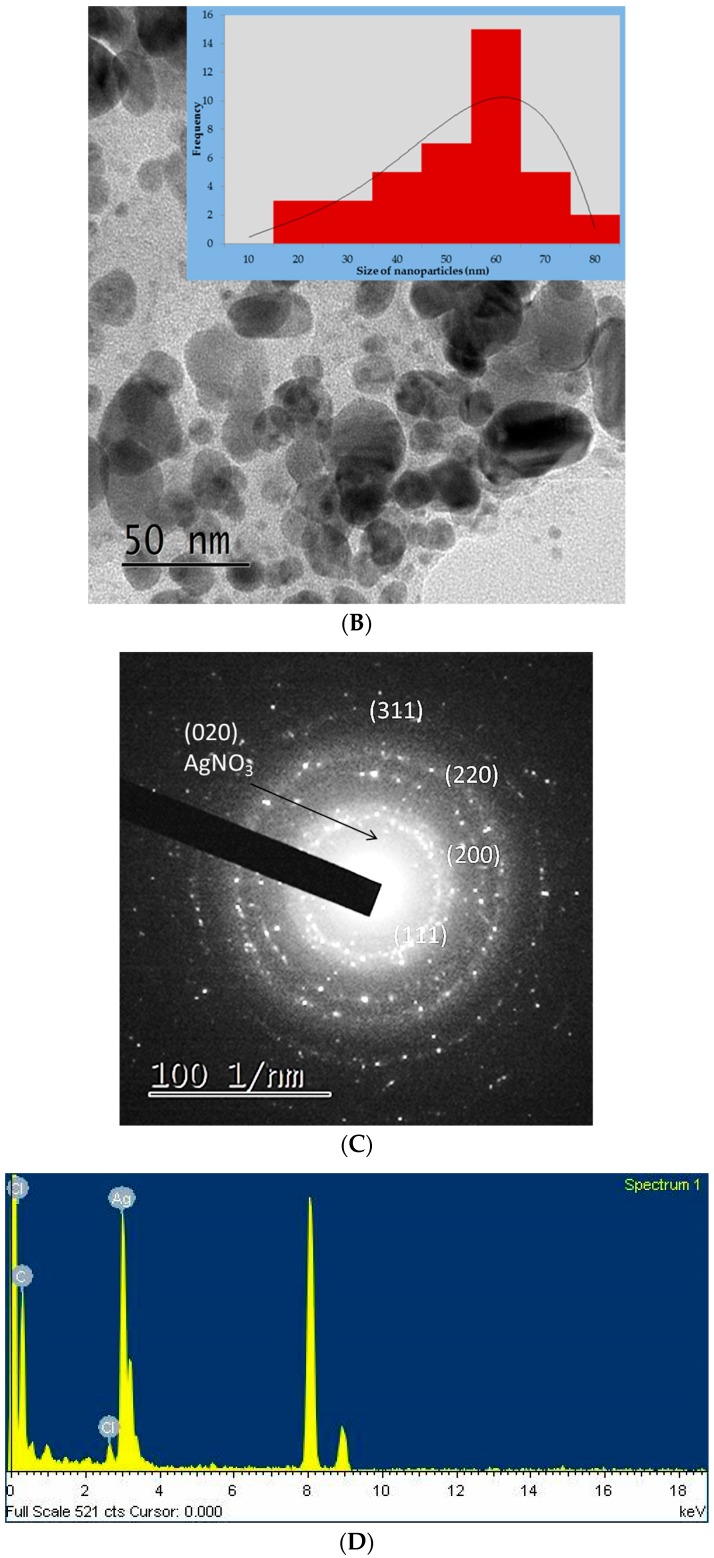
(**A**) SEM image at 500 nm resolution; (**B**) TEM image of DSAgNPs at 50 nm resolution (inset: histogram showing the particle size distribution); (**C**) SAED patern of DSAgNPs; (**D**) EDX spectra of DSAgNPs.

The SEM results indicated the size of synthesized nanoparticles was in a range of 20–72 nm, with an average size of 64 nm (Figure 2A).

**Figure 3:**
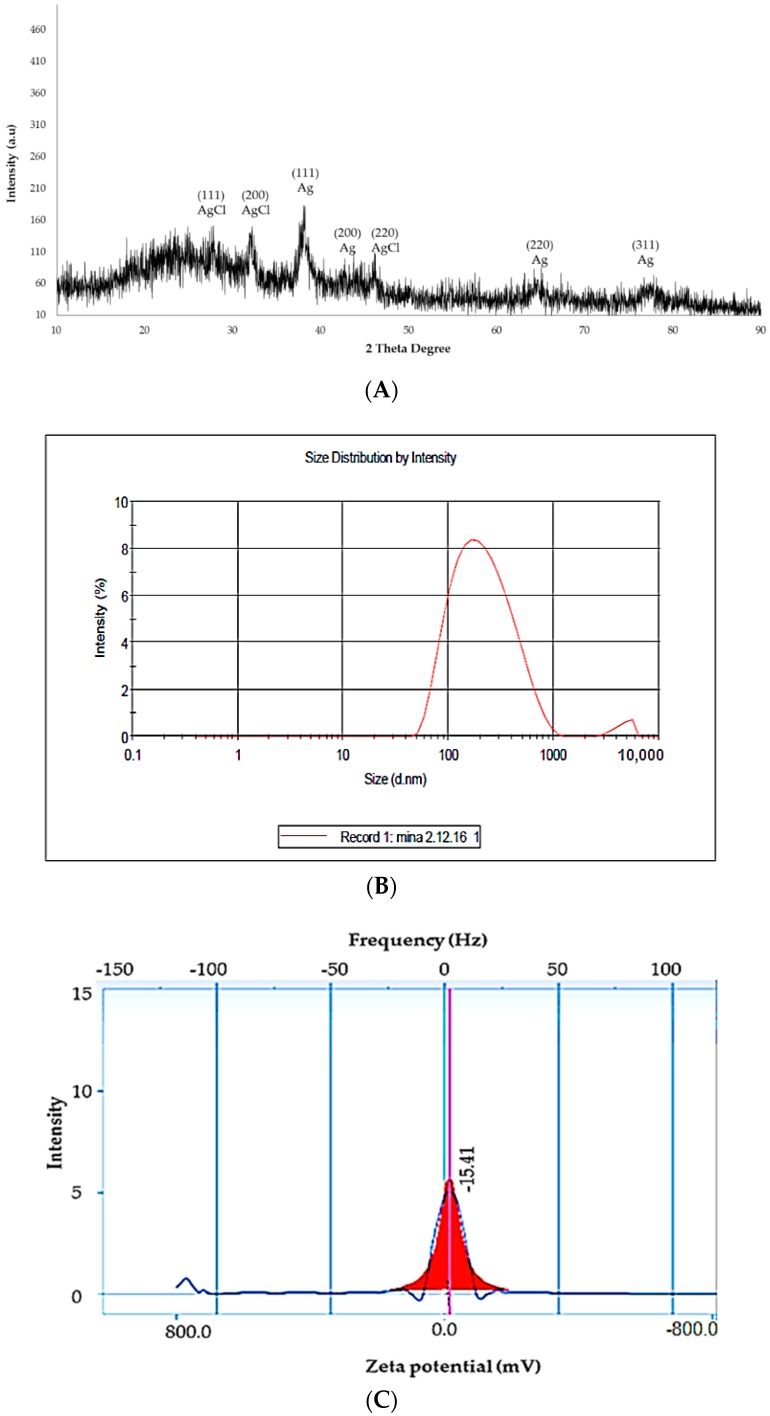
(**A**) XRD pattern of DSAgNPs, (**B**) Size distribution of DSAgNPs obtained from dynamic light scattering, (**C**) Zeta potential of DSAgNPs.

Moreover, the SEM image clearly showed polydisperse spherical and rod-shaped particles, however, the TEM images showed that the particle size ranged from 20−75 nm, with an average size of 60 nm (Figure 2B) and SAED of DSAgNPS is shown in Figure 2C. The SAED confirmed the face-centered cubic (fcc) structure of metallic silver, and it also contains some extra rings [secondary phase (020)] which indicates the presence of unreduced silver nitrate. Figure 2D shows the EDX spectrum of biosynthesized DSAgNPs which confirms the presence of elemental silver signals. Besides a high intensity peak, a few tiny weak peaks of Cl and Cr were also observed in EDX spectrum owing to biomolecules bonded onto the biosynthesized DSAgNPs. Five prominent diffraction peaks were observed in XRD pattern (Figure 3A), at 2θ = 38.18°, 44.54°, 64.28° and 77.38° which corresponds to (111), (200), (220) and (311) Bragg’s reflections of the face-centered cubic (fcc) structure of metallic silver, respectively. Moreover, the peaks observed in the pattern were also found to be in good agreement with reference of fcc structure from Joint Committee of Powder Diffraction Standard (JCPDS) Card No-087-0720 and the SAED of DSAgNPs (Figure 2C). Also another three distinct diffraction peaks at 2θ = 28.10°, 32.52° and 46.53° which corresponds to (111), (200) and (220) lattice planes of face centred cubic (fcc) structure matched to silver chloride nanoparticles (JCPDS file No.: 85-1355). AgCl is a common phase in green synthesis of silver nanoparticles [32]. The broadening of signals of pattern evinces that the products are nanosized.

The DLS analysis was carried out for determination of hydrodynamic diameters of DSAgNPs whereby the mean hydrodynamic diameter of DSAgNPs was found to be 176.5 d.nm with intercept 0.809, and a low polydispersity index (PDI) of 0.290 (Figure 3B) was observed. A higher average size value of nanoparticles was also observed through DLS analysis as compared to SEM and TEM analysis which could be attributed to agglomeration of the nanoparticles.

The DLS technique measures the hydrodynamic radii of the nanoparticles which include the solvent layer and capping at the interface. Determination of zeta potential (−15.42 mV) confirmed the stability of nanoparticles (Figure 3C). The negative potential value of DSAgNPs reflects the presence of bio-organic components in the extract, as capping agent. The value also indicated that synthesized DSAgNPs were moderately stable [33].

The explicit mechanism behind biological extract assistance in the synthesis of nanoparticles is yet to be elucidated [34]. However, phytochemicals and plant-derived polysaccharides, viz. cellulose, dextran and starch, have been reported to exert dual roles, as reducing as well as capping agents, in plant-based synthesis of nanoparticles [35]. The possible groups responsible for the interaction between capping agents and DSAgNPs were confirmed by FTIR. The FTIR spectrum of DSAgNPs showed sharp intense peaks at 3580 cm^−1^ (phenolic OH stretching), 3436 cm^−1^ (aromatic N–H stretching), 3312 cm^−1^ (OH stretching of phenol and alcohol), 1630 cm^−1^ (amide I bond of amino acids due to carbonyl stretch), 1515 cm^−1^ (aromatic ring stretching), 1375 cm^−1^ (C–C bond of aromatic ring or amide group-II), 1316-1285 cm^−1^ (aromatic secondary amine CN stretching) and 650 cm^−1^ (C–H of alkynes). The results clearly indicate that the O–H, C–C and C–N vibration stretches in DSAgNPs could be due to the presence of saccharides and amino acids in the extract of durian seeds which might be involved in the formation of DSAgNPs by acting as capping and stabilizing agents.

The stability of the synthesized DSAgNPs in aqueous dispersion was evaluated based on the changes in absorption maxima of SPR band, over a period of 120 days. The SPR bands remained almost symmetrical within the tested period. Moreover, no significant shift in the surface plasmon absorption peak was observed, which indicates that the biosynthesized DSAgNPs were not aggregated during the storage period, under ambient conditions.

### 2.4. Antimicrobial Activity of DSAgNps

The synthesized DSAgNPs showed moderate antibacterial action on the tested bacterial pathogens. The minimum inhibitory concentration (MIC) of DSAgNPs against *S. aureus* and *S. typhimurium* was found to be 4 mg/mL, and MIC against *E. coli*, *B. subtilis* and *S. typhi* was found to be 2 mg/mL. The minimum bactericidal concentration (MBC) of DSAgNPs against *E. coli*, *B. subtilis*, *S. typhimurium* and *S. typhi* was found to be 4 mg/mL (Table S1). Based on the results obtained (Table 1), it can be asserted that the newly synthesized silver nanoparticles exhibit potential antimicrobial activities against the tested pathogens. The maximum antibacterial activity was observed against *S. typhimurium*, *S. heamolyticus* and *S. aureus*, followed by *B. subtilis*, *E. coli* and *S. typhi*. DSAgNPs exhibited better antibacterial activities against all tested bacterial strains as compared to AgNO_3_ and durian seed aqueous extract alone. However, DSAgNPs expressed lesser inhibitory activities as compared to the reference drug, gentamycin, against any of the tested bacterial strains which probably due to the higher concentration of gentamycin (1 mg/mL). The study results are analogous with other studies reported by fellow researchers [36,37,38,39,40].

The mechanism of the bactericidal effect of silver nanoparticles is not well-known. Silver nanoparticles may attach to the surface of cell membrane and eventually this may disrupt power functions, viz. permeability and respiration of bacteria. The particles bind to the bacteria based on the availability of surface area for interaction. Moreover, smaller particles tend to possess larger surface area which will exhibit more bactericidal effect as compared to larger particles [36]. Morones et al., have demonstrated the presence of silver nanoparticles not only cell membrane surface but also inside the bacterium [41]. Additionally, Panacek et al., also reported that nanoparticles could release silver ions which may complement the antimicrobial properties of silver nanoparticles [36].

### 2.5. Photocatalytic Activity

The dye degradation of MB under sunlight irradiation was performed to assess the photocatalytic activities of biosynthesized DSAgNps. The dye degradation was detected by a gradual change in the solution color from deep blue to colorless.

**Figure 4:**
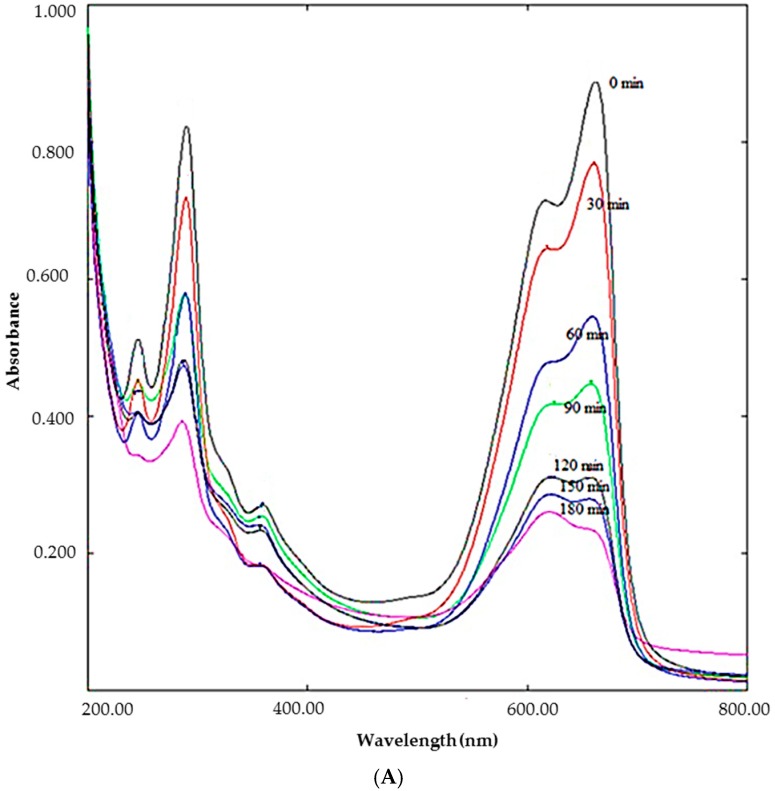

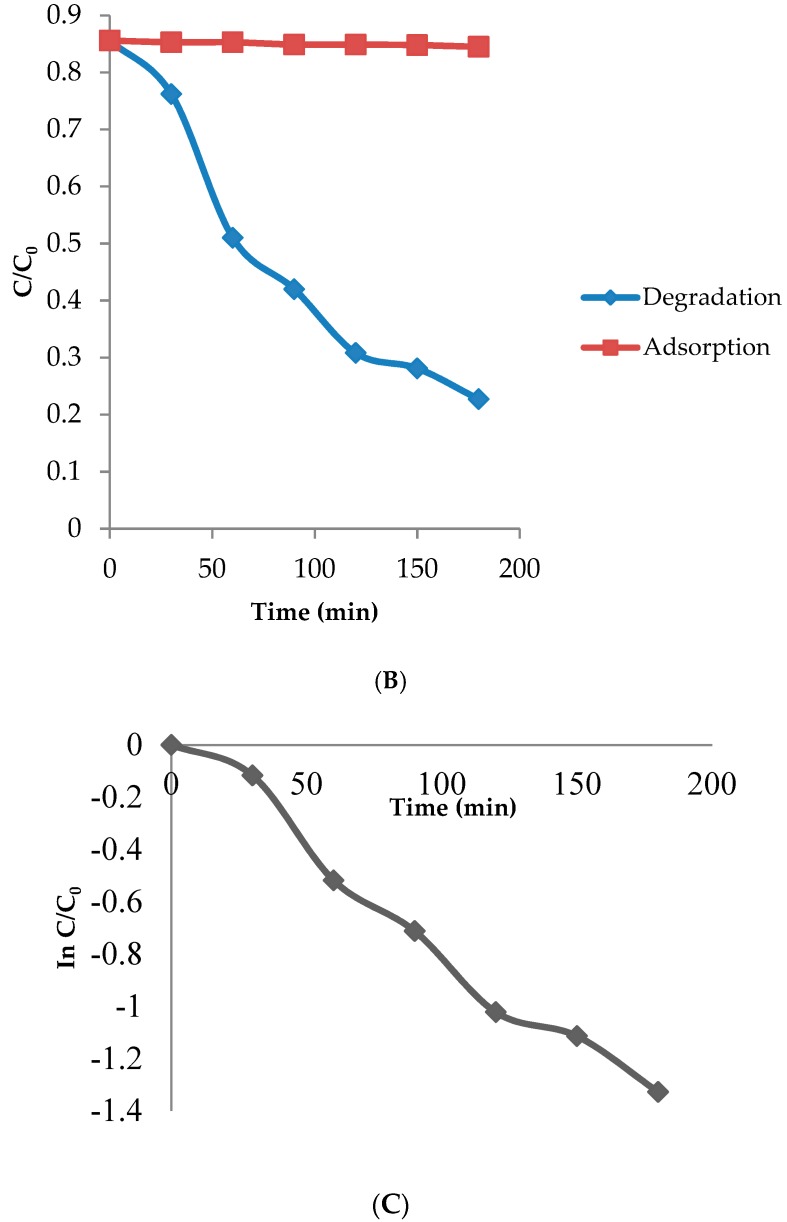
(**A**) Degradation of methylene blue under solar irradiation in the presence of DSAgNPs, (**B**) Photocatalytic degradation efficiency of DSAgNPs (**C**) Pseudo-First order kinetics.

The decrease of peak intensity at 662 nm during 3 h exposure of MB to sunlight in the presence of DSAgNPs displayed in Figure 4A indicates the photocatalytic degradation efficiency of DSAgNPs. Photocatalytic degradation efficiency of DSAgNPs was represented as the variation in MB degradation with time (C/C_0_, where C_0_ is the initial concentration of MB and C is the concentration of dye solution after “t” min under sunlight irradiation) in the presence and absence of photocatalyst (Figure 4B). The degradation was found to be 73.49% in 180 min. Additionally, the results fitted pseudo-first order kinetics (Figure 4C) and the degradation reaction rate was identified as −K = 0.0077 suggesting DSAgNPs are promising degrading agents for MB.

### 2.6. Cytotoxicity against Artemia salina

The acute toxicity of toxic materials such as pesticides and heavy metals is evaluated by utilizing *Artemia* species [42,43,44,45] As these provide distinctive advantages such as low cost, ease of culture, high offspring production, short life span, year-round availability and no requirements for feeding during assay process an extensive range of *Artemia*-based standard bioassays have been developed [45,46].

**Figure 5:**
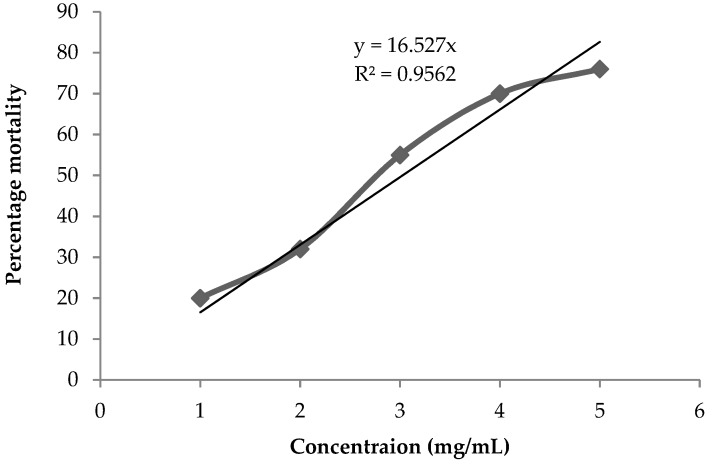
Cytotoxic effect of DSAgNPs against *Artemia salina.*

In this study, we have evaluated the cytotoxicity of DSAgNPs against brine shrimp. The brine exposures to DSAgNPs were conducted in the absence of food. The positive control (5 mg/mL of AgNO_3_) indicated about 3% mortality in 24 h which clearly validated that the experimental mortalities were not due to the deprivation from food. Concurrently, the effect of durian seed extract on survival of brine shrimp larvae was also evaluated for 24 h, during which it did not show any effect. The highest mortality of 76.0% was observed at 5 mg/mL and the calculated LC_50_ value of DSAgNPs was found to be 3.03 mg/mL (3030 mg/L). This indicated a direct proportional relationship between the concentration of DSAgNPs and lethality. Figure 5 illustrates the % mortality rates of DSAgNPs at different concentrations.

Arulvasu et al. investigated the cytotoxic effect of chemically synthesized silver nanoparticles against *Artemia* and stated that the LC_50_ value was found to be around 10 nM (10700 mg/L) concentration [47] which is comparable with the present study results. Interestingly, our cytotoxicity study results showed less cytotoxic effect against *Artemia* than the reported result of 331.08 mg/L of neem leaf extract mediated silver nanoparticles reported by Avinash et al. [48].

Moreover, Phull et al. reported a LC_50_ of 33920 mg/L for green synthesized silver nanoparticles from crude extract of *Bergenia ciliate* against brine shrimp [49] which is less toxic as compared to the DSAgNPs synthesized in this study.

Since DSAgNPs showed enhanced lethal toxicity against *Artemia* in the present study, they could be further tested for anti-tumor activities and may be further used in the management of cancer along with other anticancer drugs as carrier and active principle ingredient. Moreover, it is safer for aquatic ecology as the synthesized DSAgNPs concentration may be less as compared to the toxic concentration of industrial waste or any environmental pollution present in the aquatic environment.

Among the advantages of this study we may mention the following. First, this study provies many advantages form the viewpoint of an environmentally friendly approach: (1) waste (durian seed) is used, which is a renewable source; (2) it is a green synthesis approach we have not used any hazardous chemicals to prepare the silver nanoparticles; (3) the application of solar irradiation as a catalyzing agent in the photochemical reaction of the extract and silver salt solution is cost free; (4) photocatalytic studies of methylene blue degradation by the phytosynthesized silver nanoparticles can be envisaged to degrade organic dyes in the textile industry, or to detoxify organic toxins in waste waters.

## 3. Materials and Methods

### 3.1. Materials

Fresh and healthy durian seeds were acquired from a local market (Sungai Petani, Kedah, Malaysia). Silver nitrate (is ≥99.0%), nutrient broth (peptone 10.0 gm/L, beef extract 10.0 gm/L, sodium chloride 5.0 gm/L, pH after sterilization 7.3 ± 0.1), Muller Hinton Agar (beef infusion form 300.0 gm/L), casein acid hydrolysate 17.5 gm/L, starch 1.5 gm/L, agar 17.0 mg/L, final pH (at 25 °C) were procured from Himedia Laboratories Pvt. Ltd. (Mumbai, India). The bacterial cultures (Gram positive—*Stapylococcus aureus* ATCC 43300, *Staphylococcus heamolyticus* ATCC 29970 and *Bacillus subtilis* ATCC 6633) and Gram negative—*Salmonella typhi* ATCC 7251, *Salmonella typhimurium* ATCC 14028 and *Escherichia coli* ATCC 25922)) were obtained from Faculty of Applied Sciences, AIMST University, Malaysia.

### 3.2. Preparation of Durian Seed Extracts

The obtained durian seeds were washed with distilled water and cut into small pieces. Seeds were soaked and rinsed with 70% methanol followed by air-drying at room temperature. About 20 g of dried durian seeds were boiled with 200 mL of deionized water for 10 min in a water bath and cooled. The extract was filtered through Whatman filter paper No. 1, and centrifuged for 15 min at 5300 rpm. The supernatant was collected and stored in an amber color bottle at 4 °C for further use.

### 3.3. Biosynthesis of DSAgNPs

AgNO_3_ solution (9 mL, 1 mM) and durian seed extracts (1 mL) were mixed in a 10 mL volumetric flask which was kept in the sunlight for 30 min. The formation of DSAgNPs was corroborated by observing the change in the solution color to brown and by spectrophotometric determination.

### 3.4. Optimization of Reaction Parameters

The bioreduction of AgNO_3_ to silver nanoparticles depends on certain important parameters, viz. time, silver nitrate concentration and durian seed extract volume needed for the reaction. However, these parameters are equally important to determine the size, shape, yield and agglomeration state of DSAgNPs. Hence, the parameters were optimized for the green synthesis of DSAgNPs by altering one parameter at a time while the other parameters were kept fixed. Aqueous dispersion of DSAgNPs was prepared and stored at ambient temperature in a 50 mL volumetric flask which was protected with aluminium foil. Sample aliquots were withdrawn repeatedly at 1 week intervals for a period of 120 days to assess the stability of the synthesized DSAgNPs by measuring changes in UV-Vis spectra.

### 3.5. Characterization of DSAgNPs

The Surface Plasmon Resonance (SPR) shift and plasmon intensity of the colloidal DSAgNPs were characterized by UV-Visible spectroscopy (model AV-1800 UV-Vis spectrophotometer, Shimadzu, Tokyo, Japan) with a wavelength range of 400–700 nm. The morphology and size of the DSAgNPs were confirmed by SEM (Hitachi, S-4300 SE, Tokyo, Japan), FE-TEM (JEM-2100F, JEOL, Tokyo, Japan), elemental composition by EDX analysis (FESEM-EDX, INCA400, Oxford-Instruments, High Wycombe, UK), and surface capping functional groups by FT-IR (FTIR-4100 spectrometer, JASCO, Tokyo, Japan) at 4000 cm^−1^ to 400 cm^−1^). The crystallinity of DSAgNPs was confirmed by XRD (DMAX-2500, Rigaku, Tokyo, Japan). The zeta potential of DSAgNPs was determined by a Zetasizer Ver. 7.03 (ELC-Z Zeta potential and particle size analyzer, Photal Otsuka Electronics, Osaka, Japan).

### 3.6. Antimicrobial Activity

Synthesized DSAgNPs were investigated for antibacterial activities against Gram positive and Gram negative bacteria by Minimum Inhibitory Concentration (MIC), Minimum Bactericidal Concentration (MBC) and disc diffusion assay.

#### 3.6.1. Minimum Inhibitory Concentration (MIC)

The MIC assay was carried out by using the broth microdilution assay. Serial dilutions of DSAgNPs was added to 48-well microtiter plates, then 0.1 mL of suspension of Muller-Hinton (MH) broth medium with the bacterial cultures adjusted to 0.5 McFarland was added to each well and the plates were incubated at 37 °C for 24 h. The MIC value was determined by detecting the lowest concentration of DSAgNPs that inhibited bacterial growth after 24 h.

#### 3.6.2. Minimum Bactericidal Concentration (MBC)

About 50 µL culture suspensions from all the wells of 48-well microtiter plates which used for MIC were inoculated in MH agar medium and the plates were incubated at 37 °C for 24 h. The MBC value was determined by detecting the concentration that killed 100% bacteria.

#### 3.6.3. Disc Diffusion Assay

The selected bacteria were inoculated in LB broth. Then sterile discs of 6 mm diameter of Whatman No. 1 filter paper impregnated with 4 mg/mL of DSAgNPs in water, gentamycin (1 mg/mL) (positive control), durian seed extract (used for synthesis of silver nanoparticles), and silver nitrate (1.5 mM) and deionized water (negative control) were placed on the agar surface. The plates were incubated for 24 h at 37 °C. The test was performed in triplicate and the zones of inhibition (ZoIs) were calculated to determine the susceptibility of bacteria.

### 3.7. The Photocatalytic Degradation of Methylene Blue

To determine the photocatalytic property of DSAgNPs, 10 mg of DSAgNPs was dispersed in 100 mL of distilled water and the dispersion was sonicated for 2 h in order to determine the photocatalytic properties of DSAgNPs. Methylene blue (MB) powder (1 mg) was added to the above aqueous DSAgNPs solution. It was stirred magnetically for 30 min in the shade and upon completion the colloidal suspension was exposed to solar irradiation. During the experiment, the average atmospheric temperature was found to be 30 °C with 3 h mean sunshine duration. Suspension samples (5 mL) were repeatedly collected from the colloidal mixer at every 30 min and scanned spectrophotometrically at 200 to 800 nm for determination of the degradation of MB. The dye degradation (%) was calculated by using the following equation:

Dye degradation (%) = [C_0_ − C_t_/C_0_] × 100

where C_0_ is the initial concentration of MB solution and C_t_ is the concentration of dye solution after “t” h under sunlight irradiation. All dye concentration absorption peaks at 662 nm were measured by UV-Vis spectroscopy [50].

### 3.8. Cytotoxicity of DSAgNPs against Artemia salina

With the help of capillary glass tubes, ten live and healthy larvae of *A. salina* were transferred from the brighter part of hatching chamber to 24 well plates containing various concentrations (1–5 mg/mL) of DSAgNPs in 1 mL of sterilized seawater. As a negative control, sterilized seawater without DSAgNPs was used and maintained at 25 °C for 24 h (16 h light and 8 h dark). After incubation, the larval mortality percentage was calculated from the number of larvae surviving in each test concentration. Assays were carried out in triplicate and LC_50_ values were determined using a regression plot.

## 4. Conclusions

In this study, silver nanoparticles were synthesized by utilizing renewable sources such as durian seeds and sunlight. As it is a green synthesis approach, the reported method meets the requirement of environmental safety. In this study we have investigated the impact of silver nanoparticles on activities against human pathogens that are major sources of bacterial infections, as catalysis for the degradation of organic dyes which are prime sources of environmental pollution, and cytotoxic activities against brine shrimp which is a preliminary test to analyze the anticancer potential of the tested compounds. Various properties of the synthesized silver nanoparticles were also characterized by UV, SEM, TEM, EDX, XRD and zeta potential. SEM and TEM images confirmed the spherical nature of the silver nanoparticles. XRD images showed the presence of silver chloride nanoparticles and EDX images indicated the presence of chlorine. The extra ring in SAED of TEM indicated the presence of unreduced silver nitrate. DSAgNPs showed antibacterial activity as well as cytotoxicity against brine shrimp. The study results indicated that the green synthesis of DSAgNPs yielded an effective nanoparticle preparation that could be used against human pathogens as a potential therapeutic agent and also for the purification of wastewater in the future. This is evident since the synthesized DSAgNPs displayed good photocatalytic activities for the degradation of methylene blue and were also found to be non-toxic. The present study suggested that greenly synthesized DSAgNPs can prevent many environmental hazards in an eco-friendly way and can also be used as therapeutic agents in the pharmaceutical industry.

## Figures and Tables

**Table 1:** Antibacterial activity of DSAgNPs.

Bacteria	Zone of Inhibition (mm)	
	Gentamycin *	DSAgNPs **
*S. typhi*	25 ± 1.32	7 ± 0.20
*S. typhimurium*	24 ± 2.91	11 ± 0.51
*E. coli*	20 ± 1.06	7 ± 0.22
*S. aureus*	23 ± 1.12	8 ± 0.13
*S. haemolyticus*	23 ± 1.82	11 ± 0.55
*B. subtilis*	25 ± 1.36	8 ± 0.34

n = 3, zone of inhibition ± SD, * 1 mg/mL, ** 4 mg/mL impregnated disc were used.

## Supplementary Materials

The following are available online, Table S1: MIC and MBC results of DSAgNPS.

## References

[1] Kim J.S., Kuk E., Yu K.N., Kim J.H., Park S.J., Lee H.J. (2007). Antimicrobial effects of silver nanoparticles. Nanomedicine.

[2] Rauwel P., Rauwel E. (2017). Emerging trends in nanoparticle synthesis using plant extracts for biomedical applications. Global J. Nanomed..

[3] Saifuddin N., Wong C.W., Nur Yasumira A.A. (2009). Rapid biosynthesis of silver nanoparticles using culture supernatant of bacteria with microwave irradiation. J. Chem..

[4] Narayanan K.B., Sakthivel N. (2011). Green synthesis of biogenic metal nanoparticles by terrestrial and aquatic phototrophic and heterotrophic eukaryotes and biocompatible agents. Adv. Colloid Interface Sci..

[5] Vigneshwaran N., Ashtaputre N.M., Varadarajan P.V., Nachane R.P., Paralikar K.M., Balasubramanyan R.H. (2007). Biological synthesis of silver nanoparticles using the fungus *Aspergillus flavus*. Mater. Lett..

[6] Ramirez I.M., Bashir S., Luo Z., Liu J.L. (2009). Green synthesis and characterization of polymer-stabilized silver nanoparticles. Colloids and Surf. B Biointerfaces.

[7] Otunola G.A., Afolayan A.J., Ajayi E.O., Odeyemi S.W. (2017). Characterization, antibacterial and antioxidant properties of silver nanoparticles synthesized from aqueous extracts of *Allium sativum, Zingiber officinale*, and *Capsicum frutescents*. Pharmacogn. Mag..

[8] Veerasamy R., Xin T.Z., Gunasagaran S., Xiang T.F.W., Yang E.F.C., Jeyakumar N., Dhanraj S.A. (2011). Biosynthesis of silver nanoparticles using mangosteen leaf extract and evaluation of their antimicrobial activities. J. Saudi Chem. Soc..

[9] Ravichandran V., Shalini S., Vasanthi S., Shaa S.A.A., Harish R. (2016). Green synthesis of silver nanoparticles using *Atrocarpus altilis* leaf extract and the study of their antimicrobial and antioxidant activity. Mat. Lett..

[10] Venugopal K., Rather H.A., Rajagopal K., Shanthi M.P., Sheriff K., Illiyas M., Rather R.A., Manikandan E., Uvarajan S., Bhaskar M. (2017). Synthesis of silver nanoparticles (Ag NPs) for anticancer activities (MCF 7 breast and A549 lung cell lines) of the crude extract of *Syzygium aromaticum*. J. Photochem. Photobiol. B Biol..

[11] Nadworny P.L., Wang J., Tredget E.E., Burrell R.E. (2008). Anti-inflammatory activity of nanocrystalline silver in a porcine contact dermatitis model. Nanomed. Nanotechnol. Biol. Med..

[12] Rogers J.V., Parkinson Y.W., Choi J.L., Speshock S.M., Hussain M. (2008). A preliminary assessment of silver nanoparticle inhibition of monkeypox virus plaque formation. Nanoscale Res. Lett..

[13] Gurunathan S., Lee K., Kalishwaralal K., Sheikpranbabu S., Vaidyanathan R., Eom S. (2009). Antiangiogenic properties of silver nanoparticles. Biomaterials.

[14] Shanker K., Mohan G.K., Ashwaq Hussain Md., Jayarambabu N., Pravallika P.L. (2017). Green biosynthesis, characterization, in vitro antidiabetic activity, and investigational acute toxicity studies of some herbal-mediated silver nanoparticles on animal models. Pharmcogn. Mag..

[15] Arancibia-Avila P., Toledo F., Park Y.S., Jung S.T., Kang S.G., Heo B.G. (2008). Antioxidant properties of durian fruit as influenced by ripening. LWT-Food Sci. Technol..

[16] Toledo F., Aranciba-Avila P., Park Y.S., Jung S.T., Kang S.G., Heo B.G. (2008). Screening of the antioxidant and nutritional properties, phenolic contents and proteins of five durian cultivars. Int. J. Food Sci. Nutr..

[17] Leontowicz H., Leontowicz M., Haruenkit R., Poovarodom S., Jastrzebski Z., Drzewiecki J. (2008). Durian (*Durio zibethinus* Murr.) cultivars as nutritional supplementation to rat’s diets. Food Chem. Toxicol..

[18] Chansiripornchai P., Pongsamart S. (2008). Treatment of infected openwounds on two dogs using a film dressing of polysaccharide extracted from the hulls of durian (*Durio zibethinus* Murr.): Case report. Thai J. Vet. Med..

[19] Jaswir I., Man Y.B.C., Selamat J., Ahmad F., Sugisawa H. (2008). Retention of volatile components of durian fruit leather during processing and storage. J. Food Process. Preserv..

[20] Apak R., Guclu K., Ozyurek M., Karademir S.E. (2004). Novel total antioxidant capacity index for dietary polyphenols and vitamins C and E, using their cupric ion reducing capability in the presence of neocuproine: CUPRAC method. J. Agric. Food Chem..

[21] Hokputsa S., Gerddit W., Pongsamart S., Inngjerdingen K., Heinze T., Koschella A. (2004). Water-soluble polysaccharides with pharmaceutical importance from durian rinds (*Durio Zibethinus* Murr.): Isolation, fractionation, characterisation and bioactivity. Carbohydr. Polym..

[22] Hameed S., Sultana V., Ara J., Ehteshamul-Haque S., Athar M. (2009). Toxicity of *Fusarium solani* strains on brine shrimp (*Artemia salina*). Zool. Res..

[23] Meyer B.N., Ferrigni N.R., Putnam J.E., Jacobsen L.B., Nichols D.E., Mclaughlin J.L. (1982). Brine shrimp: A convenient general bioassay for active plant constituents. Planta Med..

[24] Smirnov V.V., Golovchenko V.V., Vityazev F.V., Patova O.A., Selivanov N.U., Selivanov O.G. (2017). The antioxidant properties of pectin fractions isolated from vegetables using a simulated gastric fluid. J. Chem..

[25] Wang X., Lu X. (2014). Characterization of pectic polysaccharides extracted from apple pomace by hot-compressed water. Carbohydr. Polym..

[26] Sharma R., Kamboj S., Khurana R., Singh G., Rana V. (2015). Physicochemical and functional performance of pectin extracted by QbD approach from *Tamarindus indica* L. pulp. Carbohydr. Polym..

[27] Lefsih K., Delattre C., Pierre G., Michaud P., Aminabhavi T.M., Dahmoune F. (2016). Extraction, characterization and gelling behavior enhancement of pectins from the cladodes of *Opuntia ficus indica*. Int. J. Biol. Macromol..

[28] Maria-Ferreira D., Da Silva L.M., Mendes D.A.G.B., de Almeida Cabrini D., Nascimento A.M., Iacomini M. (2014). Rhamnogalacturonan from *Acmella oleracea* (L.) R.K. Jansen: Gastroprotective and ulcer healing properties in rats. PLoS ONE.

[29] Mulvaney P. (1996). Surface plasmon spectroscopy of nanosized metal particles. Langmuir.

[30] Rastogi L., Arunachalam J. (2011). Sunlight based irradiation strategy for rapid green synthesis of highly stable silver nanoparticles using aqueous garlic (*Allium sativum*) extract and their antibacterial potential. Mater. Chem. Phys..

[31] Nadezhda V.I., Natalya N.T., Lydmila A.E., Vasilyi A.B. (2012). The study of the reaction of pectin-Ag(0) nanocomposites formation. Int. J. Carbohydr. Chem..

[32] Awwad A.m., Salem N.M., Ibrahim Q.M., Abdeen A.O. (2015). Phytochemical fabrication and characterization of silver/ silver chloride nanoparticles using *Albizia julibrissin* flowers extract. Adv. Mater. Lett..

[33] Parameshwaran R., Kalaiselvam S., Jayavel R. (2013). Green synthesis of silver nanoparticles using Beta vulgaris: Role of process conditions on size distribution and surface structure. Mater. Chem. Phys..

[34] Singh G., Babele P.K., Shahi S.K., Sinha R.P., Tyagi M.B., Kumar A. (2014). Green synthesis of silver nanoparticles using cell extracts of *Anabaena doliolum* and screening of its antibacterial and antitumor activity. J. Microbiol. Biotechnol..

[35] Park Y. (2014). A new paradigm shift for the green synthesis of antibacterial silver nanoparticles utilizing plant extracts. Toxicol. Res..

[36] Panacek A., Kvytek L., Prucek R., Kolar M., Vecerova R. (2008). Silver colloid nanoparticles: Synthesis, characterization, and their antibacterial activity. J. Phys. Chem. B.

[37] Patil Shriniwas P., Kumbhar Subhash T. (2017). Antioxidant, antibacterial and cytotoxic potential of silver nanoparticles synthesized using terpenes rich extract of *Lantana camara* L. leaves. Biochem. Biophys. Rep..

[38] Logeswari P., Silambarasan S., Abraham J. (2015). Synthesis of silver nanoparticles using plants extract and analysis of their antimicrobial property. J. Saudi Chem. Soc..

[39] Lee J.H., Lim J.M., Velmurugan P., Park Y.J., Park Y.J., Bang K.S., Oh B.T. (2016). Photobiologic-mediated fabrication of silver nanoparticles with antibacterial activity. J. Photochem. Photobiol. B Biol..

[40] Banerjee P., Satapathy P., Mukhopahayay A., Das P. (2014). Leaf extract mediated green synthesis of silver nanoparticles from widely available Indian plants: Synthesis, characterization, antimicrobial property and toxicity analysis. Bioresour. Bioprocess..

[41] Morones J.R., Elechiguerra J.L., Camacho A., Holt K., Kouri J. (2005). The bactericidal effect of silver nanoparticles. Nanotechnology.

[42] Sorgeloos P. (1980). Availability of reference Artemia cysts. Mar. Ecol. Prog. Ser..

[43] Gajbhiye S.N., Hirota R. (1990). Toxicity of heavy metals to brine shrimp Artemia. J. Indian Fish Assoc..

[44] Nunes B.S., Carvalho F.D., Guilhermino L.M., Van Stappen G. (2006). Use of the genus Artemia in ecotoxicity testing. Environ. Pollut..

[45] Kokkali V., Katramados I., Newman J.D. (2011). Monitoring the effect of metal ions on the mobility of *Artemia salina* nauplii. Biosensors.

[46] Vanhaecke P., Persoone G., Claus C., Sorgeloos P. (1981). Proposal for a short-term toxicity test with *Artemia nauplii*. Ecotoxicol. Environ. Safety.

[47] Arulvasu C., Jennifer S.M., Prabhu D., Chandhirasekar D. (2014). Toxicity effect of silver nanoparticles in brine shrimp *Artemia*. Sci. World J..

[48] Avinash B., Supraja N., Santhi Priya Ch., Prasad T.N.V.K.V., Alpha Raj M. (2017). Synthesis characterization and evaluation of the antimicrobial activity of neem leaf extract-mediated silver nanoparticles. Int. J. Pure Appl. Biosci..

[49] Phull A.-R., Abbas Q., Ali A., Raza H., Kim S.J., Zia M. (2016). Antioxidant, cytotoxic and antimicrobial activities of green synthesized silver nanoparticles from crude extract of *Bergenia ciliate*. Future J. Pharm. Sci..

[50] Prakash S., Ahila N.K., Sri Ramkumar V., Ravindran J., Kannapiran E. (2015). Antimicrofouling properties of chosen marine plants: An eco-friendly approach to restrain marine microfoulers. Biocatal. Agric. Biotechnol..

