# Supplementary material for: Phyto-Mediated Photo Catalysed Green Synthesis of Silver Nanoparticles Using Durio Zibethinus Seed Extract: Antimicrobial and Cytotoxic Activity and Photocatalytic Applications
Source: Molecules. 2018 Dec 13;23(12):3311. doi: 10.3390/molecules23123311 (PMC6320879; doi:10.3390/molecules23123311)
Supplement: Supplementary file 1 [file molecules-23-03311-s001.pdf]

Supplementary material

Phyto-Mediated Photo Catalysed Green Synthesis of Silver Nanoparticles Using *Durio Zibethinus* Seed Extract: Antimicrobial and Cytotoxic Activity and Photocatalytic Applications

Samuggam Sumitha [1], Sethu Vasanthi [2], Sivadasan Shalini [3,4], Suresh V. Chinni [1], Subash C.B. Gopinath [5], Periasamy Anbu [6], Mohammed Baidi Bahari [4], Rajak Harish [7], Sathasivam Kathiresan [1] and Veerasamy Ravichandran [4,*]

[1] Faculty of Applied Science, AIMST University, Semeling 8100, Bedong, Kedah, Malaysia; sumithasamuggam@gmail.com (S.S.), v_suresh@aimst.edu.my (S.V.C), kkdjps@gmail.com (S.K)
[2] Faculty of Engineering, The University of Nottingham, Semenyih 43500, Selangor, Malaysia; Vasanthi.Sethu@nottingham.edu.my
[3] Department of Pharmacy Practice, KMCH College of Pharmacy, Coimbatore 641035, India; shaliniravichandran11@gmail.com
[4] Faculty of Pharmacy, AIMST University, Semeling 8100, Bedong, Kedah, Malaysia; baidi_bahari@aimst.edu.my
[5] School of Bioprocess Engineering, Universiti Malaysia Perlis, 02600 Arau, Perlis, Malaysia; subash@unimap.edu.my
[6] Department of Biological Engineering, Inha University, Incheon 402-751, Korea; anbu25@yahoo.com
[7] SLT Institute of Pharmaceutical Sciences, Guru Ghasidas University, Bilaspur 495009, India; harishdops@yahoo.co.in
* Correspondence: phravi75@rediffmail.com; ravichandran_v@aimst.edu.my; Tel.: +6-04-4298000-1278

Table S1. MIC and MBC results of DSAgNPS.

	No. of colonies							
**Bacteria**	**Positive**	**Negative**	**4 mg/mL**	**2 mg/mL**	**1 mg/mL**	**0.5 mg/mL**	**0.25 mg/mL**	**0.125 mg/mL**
S. Typhi	0	TNTC	0	15	TNTC	TNTC	TNTC	TNTC
S. typhimurium	0	TNTC	0	418	TNTC	TNTC	TNTC	TNTC
E. coli	0	TNTC	0	3	TNTC	TNTC	TNTC	TNTC
S. aureus	0	TNTC	91	TNTC	TNTC	TNTC	TNTC	TNTC
S. haemolyticus	0	TNTC	194	493	TNTC	TNTC	TNTC	TNTC
B. subtilis	0	TNTC	4	62	TNTC	TNTC	TNTC	TNTC

TNTC - Too numerous to count.
